# Kick-Starting Wound Healing: A Review of Pro-Healing Drugs

**DOI:** 10.3390/ijms25021304

**Published:** 2024-01-21

**Authors:** Bethany L. Patenall, Kristyn A. Carter, Matthew R. Ramsey

**Affiliations:** Department of Dermatology, Brigham and Women’s Hospital, Harvard Medical School, Boston, MA 02115, USAkcarter15@bwh.harvard.edu (K.A.C.)

**Keywords:** chronic wounds, diabetes, wound healing, epithelization, macrophages, biomaterials

## Abstract

Cutaneous wound healing consists of four stages: hemostasis, inflammation, proliferation/repair, and remodeling. While healthy wounds normally heal in four to six weeks, a variety of underlying medical conditions can impair the progression through the stages of wound healing, resulting in the development of chronic, non-healing wounds. Great progress has been made in developing wound dressings and improving surgical techniques, yet challenges remain in finding effective therapeutics that directly promote healing. This review examines the current understanding of the pro-healing effects of targeted pharmaceuticals, re-purposed drugs, natural products, and cell-based therapies on the various cell types present in normal and chronic wounds. Overall, despite several promising studies, there remains only one therapeutic approved by the United States Food and Drug Administration (FDA), Becaplermin, shown to significantly improve wound closure in the clinic. This highlights the need for new approaches aimed at understanding and targeting the underlying mechanisms impeding wound closure and moving the field from the management of chronic wounds towards resolving wounds.

## 1. Introduction

### 1.1. Normal Wound Healing

**Figure 1:**
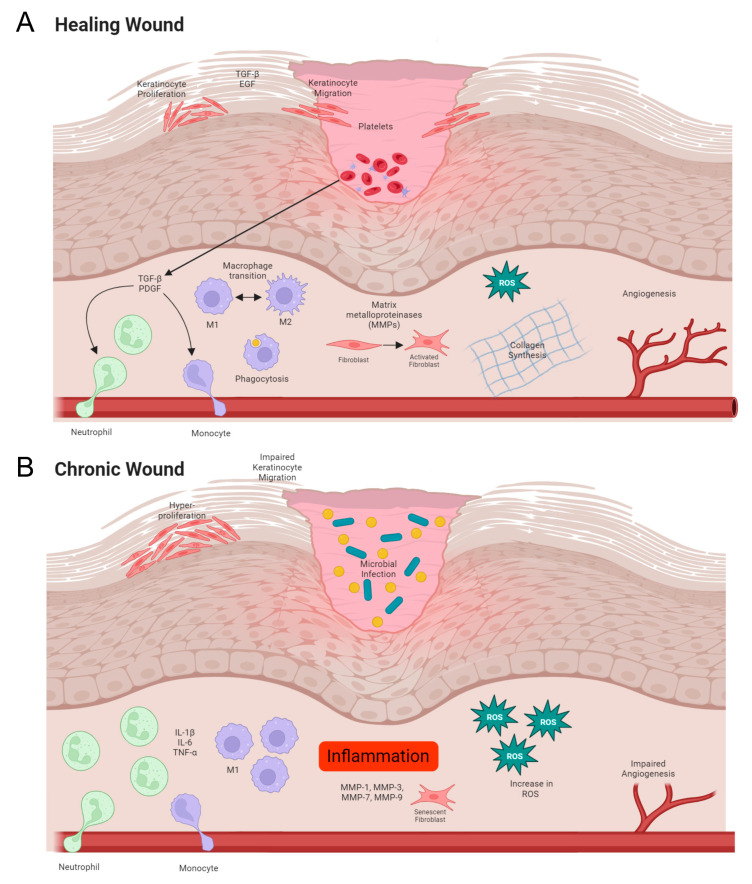
Normal versus chronic wound healing. (**A**) In normal wounds, there is an orderly progression from hemostasis to inflammation, proliferation/repair and, finally, remodeling. (**B**) Chronic wounds demonstrate increased inflammation, reduced keratinocyte migration associated with hyperproliferation and the presence of bacterial biofilms.

Wound healing is a complex process classified into four defined stages: hemostasis, inflammation, proliferation/repair and remodeling (Figure 1A). Immediately after injury, hemostasis occurs through the formation of a fibrin and platelet plug which trigger a coagulation cascade to stop the bleeding at the site of injury and promote recruitment of cells from the surrounding tissue and bloodstream. The fibrin plug, from platelet-derived fibrinogen, acts as a matrix for fibroblasts and macrophages [1]. Damage to endothelial cells exposes collagen which stimulates platelets to undergo activation, adhesion, and aggregation. Platelets produce chemotactic factors including transforming growth factor-β (TGF-β) and platelet-derived growth factor (PDGF). These growth factors attract neutrophils, macrophages and fibroblasts, which are essential for the initiation and completion of the inflammatory and proliferative stages of healing. In addition, changes in osmolarity and an increase in hydrogen peroxide contribute to leukocyte recruitment to the wound site [2,3].

The inflammatory phase starts within minutes of wound formation when neutrophils adhere to the endothelium. Neutrophils use collagen and elastase to facilitate migration into the extracellular space where they degrade matrix proteins, phagocytose microbes, and further attract additional neutrophils as well as macrophages. Macrophages play a key role in the acute healing process and are the predominant cell type during the inflammatory stage. Macrophage differentiation exists on a spectrum, commonly delineated as either M1 leaning or M2 leaning. The phenotype of macrophages changes as wounds progress through the stages of healing, towards a resolved healed response. During the early stages of wound healing, macrophages differentiate into an M1 phenotype, which infiltrates the wound site and removes bacteria, debris, and dead cells. Then, as the wound begins to repair the macrophage population transitions to an M2 phenotype which promotes resolution of inflammation and enables migration and proliferation of fibroblasts and keratinocytes and rebuilding of the tissue architecture [4,5].

The proliferation phase begins within 24 h of wounding and comprises fibroplasia, granulation, epithelialization and angiogenesis. The fibrin matrix created during hemostasis enables keratinocyte migration from the wound edge and hair follicles across the keratinocytes within the wound bed. In addition to TGF-β and Interleukin-6 (IL-6), the production of key molecules Epidermal Growth Factor (EGF) and Tumor Necrosis Factor-α (TNF-α) are essential to balance keratinocyte proliferation and migration [6]. Interestingly, disruption of the epithelium generates a directional electrical field, which also helps orient keratinocytes for directed migration [7]. Angiogenesis is induced by the presence of Vascular Endothelial Growth Factor (VEGF), which is upregulated by low oxygen tension [8]. Endothelial cells are recruited and stimulated to proliferate by VEGF, which induces smooth muscle cell migration [9].

Fibroblasts migrate to the wound site between 48–72 h post wound and are integral for dermal matrix repair. Fibroblasts produce structural proteins including elastin, matrix metalloproteinases (MMPs) and collagen family members. MMPs degrade the fibrin plug which was formed during hemostasis and facilitate fibroblast movement. Collagen is present 48–72 h post wounding and is at peak secretion between 5 and 7 days [10]. Remodeling of the wound takes weeks to years. Wound contraction begins around 5 days post wounding as fibroblasts change into myofibroblasts which are predominantly actin producers. MMPs and MMP inhibitors reorganize type III collagen into a strong network of type I collagen. Collagen reaches 20% of its tensile strength after ~3 weeks and 80% strength in 12 months, during which the skin is fragile and prone to re-wounding [1].

### 1.2. Impaired Wound Healing in Chronic Wounds

Chronic wounds are defined by their failure to progress through the stages of wound healing in a regulated and timely fashion. Wound healing takes between four to six weeks, whereas chronic wounds can take significantly longer or can fail to heal entirely [11]. This loose definition is largely a result of the heterogeneity in chronic wound etiology. Wounds vary greatly in location, size, and host factors. Chronic wounds broadly affect the adult population and the impact is exacerbated by comorbidities such as diabetes, cardiovascular disease, venous/arterial insufficiency and/or lack of mobility [12]. Chronic wounds can be further subclassified into arterial and venous ulcers, pressure ulcers and diabetic ulcers.

It is estimated that approximately 2% of the population will experience a chronic wound in their lifetime. This translates to 5.7 million people in the United States and an annual cost of around $20 billion. In addition to this economic burden, chronic wounds precede 85% of all amputations, with diabetic ulcers responsible for 70% of all lower limb amputations. Unfortunately, the 5-year mortality rate following amputation is between 40–70% [13], demonstrating the importance of effectively managing these wounds. Chronic wounds tend to be treated as a co-morbidity of other conditions by clinicians from a range of specialties including dermatology, podiatry, and geriatrics. Clinicians often lack specialized training in the diagnosis and treatment of wounds as it is not a defined specialty, leading to variations in treatment and wound management.

Chronic wounds generally stagnate between the inflammatory and proliferation stages, failing to reduce inflammation and rebuild the tissue architecture (Figure 1B). Often there is an increase in acute inflammatory cells such as macrophages and neutrophils, as well as cytokines including Interleukin-1β (IL-1β) and TNF-α and an absence of cellular growth and keratinocyte migration over the wound [13]. In addition, oxidative stress is known to impair the healing process. Reactive oxygen species (ROS)-mediated transcription can lead to sustained pro-inflammatory cytokine secretion and induction of MMPs specifically MMP-1, -3, -7 and -9. Within chronic wounds it is known that MMPs have higher protease activity which contributes to the degradation of the extracellular matrix (ECM), thus preventing healing. Excessive ROS can degrade the extracellular matrix and impair dermal fibroblast and keratinocyte function both directly and indirectly through the activation of proteolysis [14,15]. While low levels of ROS are required for intracellular signaling and defense against pathogens and can increase the rate of wound healing, higher concentrations can prevent keratinocyte migration impeding healing [16].

One of the major impediments to wound healing is infection, which contributes to wound chronicity. Bacteria colonize the wound and irreversibly bind to the wound surface, forming complex communities of bacteria known as biofilms. These chronic, infected wounds are significantly harder to treat owing to their thick extracellular matrix and high prevalence of antimicrobial resistance. In addition, biofilms are hard to remove through physical methods such as sharps debridement [17,18]. Biofilms are known to be present in at least 60% of all chronic wounds and there is evidence that they form as early as 10 h post wounding [19]. It is thus unsurprising that many research efforts focus on antimicrobial therapies including therapeutic release hydrogels and novel methods of debridement.

## 2. Current Therapeutics for Wound Healing

**Figure 2:**
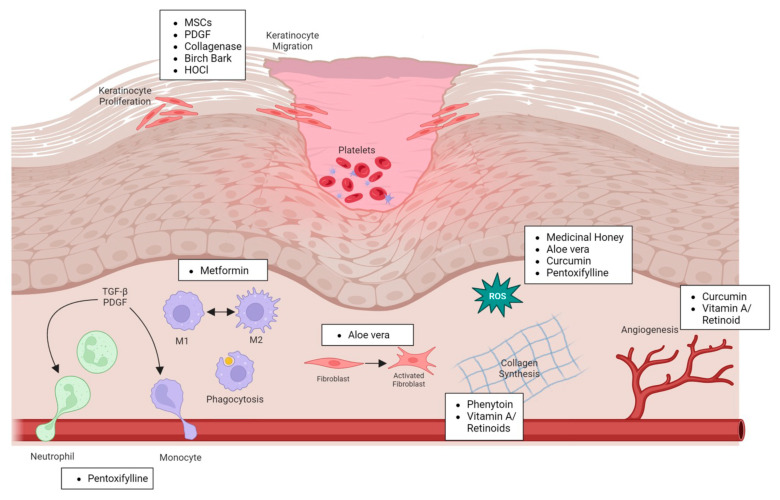
Cell types affected by different pro-healing treatments.

The care of chronic wounds has seen major advancements over the years, particularly with improved surgical wound bed preparation [20,21,22] and better wound dressings [23]. Wound dressings incorporating novel biomaterials [24], altering fluid balance [25] and modifying the pH of the wound environment [26,27,28] have greatly improved outcomes for chronic wound patients. Importantly, chronic wounds are often a result of underlying pathology, and advances in the treatment of diabetes [29,30] and venous insufficiency [31] are some of the best ways to reduce the burden of chronic wounds. Here, we examine the progress made in developing therapeutics designed to improve the healing of chronic wounds and their proposed mechanisms of action (Figure 2).

### 2.1. Natural Products

#### 2.1.1. Antibiotics

Antibiotics are a front-line therapeutic within wound care and, while their primary use is to eradicate microbial infection, there are several studies that have shown that they may have some pro-healing efficacy. Primarily, antibiotics kill or prevent the growth of the micro-organisms that cause infection and prevent it from spreading and worsening its effects [32]. Through the removal of micro-organisms, it is thought that antibiotics indirectly enable wound healing to occur more quickly [33]. While there is little evidence in the literature of antibiotics directly affecting wound healing, several studies have shown the positive impacts of antibiotics on healing time. Hwang et al. found that a gentamicin-loaded hydrogel wound dressing significantly increased wound healing in a non-infectious murine wound model compared to vehicle control dressing and untreated control wounds [34]. Further, Lin et al. showed a separate gentamicin hydrogel resulted in an increase in wound healing within a rat wound model [35]. Interestingly, Li et al. showed that a ciprofloxacin-releasing wound dressing significantly increased wound healing when compared to vehicle control in a murine wound model, the group also showed that in response to ciprofloxacin, CD34 expression was increased resulting in a vascular endothelial cell proliferation and migration to the wound area where they participate in the regeneration of blood capillaries and promote healing [36].

#### 2.1.2. Silver

Silver dressings are widely used within wound care, and silver is known to be antimicrobial, clearing bacterial contamination and thus enabling wound healing to occur more easily. There is extensive evidence of silver’s utility in infected open wounds [37,38,39,40]. However, despite the antimicrobial efficacy of silver, it is also toxic to fibroblasts when present in high concentrations and thus can lead to impaired wound healing. Silver sulfadiazine (SSD) is a topical cream/ointment used within wound care, but interestingly it has been shown to lead to slower epithelialization in multiple randomized control trials [41]. It is hypothesized that the heavy metal poisoning induced by SSD, which gives rise to its favorable antimicrobial properties, can also have a toxic effect on keratinocytes causing the observed, slowed reepithelization [42]. Further, systemic toxicity of silver can occur through absorption of silver through the skin and manifests as irreversible grey skin discoloration and loss of night vision. Luckily, this is rare, with only 16 recorded cases in the U.S., as serum silver is rapidly excreted in urine and feces [43]. Localized silver toxicity occurs more commonly owing to the cytotoxicity of silver ions against keratinocytes and fibroblasts. Toussaint et al. showed that Mepilex Ag had a slower healing time in a non-infected porcine burn model than antibiotic ointment [44]. Furthermore, Innes et al. [45] showed that in skin graft donor sites the silver-containing surgical dressing Acticoat^®^ was found to significantly delay epithelialization when compared to an occlusive dressing [30]. While silver has utility within infected wound care once a wound is clean silver-free dressings should be used owing to their detrimental effects on epithelization [41]. It should be noted that the impact of silver on wound healing varies on mode of delivery, release rate, concentration, and exposure. For example, it has been shown that nanocrystalline silver is the most potent delivery system and resulted in the greatest acceleration in wound re-epithelialization and multiple studies have shown that silver nanoparticles (AgNPs) have been shown to increase wound healing [46,47,48].

#### 2.1.3. Medicinal Honey

Honey has been used in medicine throughout history and was first used by the Egyptians in surgical dressings to facilitate wound healing. Within wound care, honey is known to be antimicrobial but it also has additional pro-healing effects [49]. Medical honey is known to upregulate pro-inflammatory cytokines TNF-α, IL-1β, IL-6 and prostaglandin E_2_ production, aiding in the inflammatory phase. Honey can also increase MMP-9 and TGF-β, contributing to the proliferative and remodeling phase [50]. One type of medicinal honey, manuka honey, is widely used in wound care. Manuka honey has a cocktail of enzymes in it, one of which is glucose oxidase, which catalyzes the oxidation of glucose to gluconic acid and H_2_O_2_. Gluconic acid results in a reduction in pH and the H_2_O_2_ is anti-bacterial. This pH change results in a reduction in protease activity at the wound site and a subsequent increase in oxygen release from hemoglobin resulting in the stimulation of fibroblast and macrophage activity. In addition, the H_2_O_2_ stimulates VEGF production. Further, flavonoids are present in honey which are ROS scavengers, neutralizing free radicles and further enhancing healing [51]. There are several Manuka honey dressings approved by the FDA varying in the proportion of Manuka honey applied to the wound. Robson et al. highlight the success of Medihoney^TM^ in their clinical setting and describe 90% of their cases of chronic wounds as successfully closed [52]. Moreover, Biglari et al. demonstrated a significant reduction in healing time with Medihoney^TM^ in patients with chronic pressure ulcers [53]. While the utility of medical honey as an antimicrobial in wounds is well understood, further study is required to understand if medical honey is physiologically driving healing as opposed to simply reducing bacterial contamination and enabling improved healing.

#### 2.1.4. Curcumin

Curcumin is a polyphenol derived from the rhizome of *Curcuma longa*, which is commonly known as turmeric. Curcumin has historically been used within herbal medicine across the globe with applications in wound care and other illnesses owing to its anti-inflammatory, antioxidant, antimicrobial and anti-cancer properties. Curcumin modulates inflammatory, proliferative and remodeling phases of wound healing [54]. It has been reported to inhibit the production of TNF-α and IL-1 via NF-ĸB signaling, which are key cytokines in mediating inflammation [55,56]. In addition, curcumin scavenges ROS, mitigating oxidative stress and increasing the production of collagen and hydroxyproline during the proliferative phase of wound healing [54,57]. Gadekar et al. [58] showed that applying transdermal curcumin patches to excisional wounds in rats promoted wound contraction and angiogenesis, resulting in reduced healing time [32]. This phenomenon was further explored in vitro by Phan et al. who used an H_2_O_2_ model of damage on human fibroblasts and keratinocytes to demonstrate successful repair after curcumin administration [59]. Curcumin also has been shown to play a role in the proliferative stage of healing. Gopinath et al. treated wounded rats with curcumin-loaded chitosan sponges and found that there was a better alignment of granulation tissue compared to a control [57]. Curcumin is thus able to accelerate the process of wound healing by shortening the inflammatory phase and aiding in proliferation and remodeling. Curcumin’s hydrophobicity results in poor oral absorption, and thus it is more commonly used for topical application [54].

#### 2.1.5. Aloe Vera

Aloe vera is derived from the cactus-like plant *Aloe barbadensis* and has been used throughout history, with its earliest use documented by Egyptians in 4000 B.C. [60,61]. Aloe vera is used to treat burns and ulcers and has been shown to reduce pain and improve healing time [62]. Aloe vera is also known to decrease TNF-α and IL-1 [63,64] and its phenolic compound content promotes ROS scavenging, reducing inflammation [65,66]. In addition, Aloe vera contains polysaccharides, such as mannose-6-phosphate, which bind and stimulate fibroblast activity and proliferation, which increases collagen production [63].

#### 2.1.6. Birch Bark

*Betula alba* (birch bark) has been used within traditional medicine across the northern hemisphere and was first used in wound care by the Native American Ojibwe tribe, who would wrap their wounds with birch bark to accelerate healing [67]. Birch bark’s healing properties have since been proven clinically using n-heptane dry extract from the outer bark of the birch; 97% of the extract is pentacyclic triterpenes [68] and the triterpene responsible for wound healing is botulin [69]. Ebeling et al. showed that triterpenes significantly increased wound healing in an ex vivo porcine healing model, demonstrating improved skin barrier and enhanced migration when applied to human keratinocytes, mediated through IL-6 and signal transducer and activator of transcription 3 (STAT3) signaling [70]. A birch bark gel bark extract (Episalvan^®^) has found accelerated re-epithelialization in partial thickness skin wounds [71] and superficial partial thickness burns [72] and has been approved for use in humans by the European Medicines Agency (EMA); however, it is yet to be approved by the United States FDA. 

### 2.2. Human-Derived Factors

#### 2.2.1. Mesenchymal Stem Cells

Mesenchymal stem cells (MSCs) are multipotent stem cells derived from the mesoderm and give rise to osteoblasts, chondrocytes, adipocytes, and reticular stroma. MSCs can be isolated from a variety of sources, such as bone marrow, umbilical cord tissue, the placenta, and adipose tissue [73]. The fundamental biological mechanism of mesenchymal stem cell-induced wound healing is thought to be due to their ability to secrete pro-regenerative cytokines [74]. MSCs modulate the immune response, through the secretion of interferon gamma (IFN-λ) and TNF-α, leading to an increase in the secretion of IL-10 and IL-4 produced by various immune cells including but not limited to macrophages, dendritic cells (DCs), and lymphocytes [75,76]. In addition, MSCs promote the formation of new vessels and extracellular matrix and mediate cell proliferation and differentiation through the secretion of VEGF, keratinocyte growth factor, MMP-9, and EGF [77]. 

There have been several trials using MSCs to treat chronic, diabetic wounds. Hashemi et al. seeded an acellular amniotic membrane with Wharton’s jelly mesenchymal stem cells and reported a reduction in wound size and time needed to heal [78]. Vojtaššák et al. applied a biodegradable collagen membrane (Coladerm) in combination with autologous MSCs from the patient’s bone marrow to the patient’s diabetic foot ulcer on days 0, 7 and 17, relative to when treatment began. By day 29, closing and healing of the wound was achieved [79]. Multiple clinical trials are ongoing, including Cell2Cure’s study “STEMFOOT” (Trial no: NCT05595681) which is an “off-the-shelf” adipose tissue-derived mesenchymal stem cell product [80]. 

Major drawbacks of MSC therapy include the “standardization” of manufacturing and quality control owing to the variation in cellular proliferation and differentiation capacity between donors. In addition, there is variation observed between subpopulations of MSCs from a single source owing to RNA production variation [81]. Further complicating this is the issue of which site to obtain MSCs, as bone marrow-derived MSCs are commonly used in cutaneous wound healing whereas adipose-derived and umbilical cord-derived cells have been used in diabetic ulcer trials [82,83].

#### 2.2.2. Macrophages

Macrophage modulation has been explored as a potential therapeutic option. Goren et al. systemically administered neutralizing monoclonal antibodies anti-TNF-α and anti-F4/80 into diabetic wound models and reported the antibodies effectively targeted and killed pro-inflammatory wound macrophages resulting in accelerated healing [84]. Danon et al. administered macrophages obtained from the blood of young healthy donors and stimulated by hypo-osmotic shock, intradermally near pressure ulcer site in elderly patients which resulted in an increased rate of healing [85]. This was further confirmed by Zuloff-Shani et al. with intradermal injection of macrophages increasing healing in both pressure and diabetic ulcers [86].

#### 2.2.3. Collagenase

Collagenase belongs to the metalloproteinase family and plays an important role in the metabolism of collagen in mammalian tissues. Skin consists of between 70–80% collagen; thus, unsurprisingly, the action of collagenase is immensely important. Collagenases are the only enzymes that can specifically cleave native collagen. In healthy wounds, endogenous collagenase breaks down necrotic tissue to enable healing to occur. However, often in non-healing wounds the underlying disease etiology such as diabetes or age may cause impaired collagenase activity, resulting in a buildup of necrotic tissue preventing healing from occurring [87]. Collagenase can therefore be used as a debridement agent, as it can break down necrotic and/or fibrotic tissues within sites of tissue damage without damaging healthy tissue, creating a more congruent wound bed for healing. Interestingly, collagenase has also been shown to increase proliferation, angiogenesis and migration within wounds [88]. 

Collagenase Santyl^®^ ointment (Smith & Nephew) is a preparation of enzymes including collagenase and non-specific proteases produced by *Clostridium histolyticum* fermentation and is approved by the FDA for clinical use [89]. Tallis et al. showed that collagenase ointment significantly improved the wound bed appearance and enhanced the rate of healing [90]. Riley et al. showed in vitro that collagenase indeed does promote keratinocyte proliferation and keratinocyte migration. Further, in vivo findings showed that collagenase increased the rate of re-epithelization and increased wound closure rate in the mini-pig wound model [91]. 

#### 2.2.4. Placental-Derived Products

Tissue derived from the placenta contains a variety of growth factors (PDGF-BB, TGFα, bFGF and EGF), cytokines (IL-4, IL-6, IL-8 and IL-10) and ECM components [92], which have been shown to contribute to wound healing. This can include dehydrated human amnion/chorion membrane (dHACM) products such as EPIFIX (MiMedx) and a dehydrated human umbilical cord (DHUC) such as EPICORD (MiMedx). In vitro studies have shown that dHACM can increase fibroblast migration and induce MSC migration. This MSC migration was also seen in a murine wound model which was treated with dHCAM [92]. As these grafts contain a complex mixture of components with biological activity, the key factors mediating these effects are not clear. However, there is ample clinical evidence for a positive effect on wound healing in patients [93,94] and this treatment is recommended by The International Working Group on the Diabetic Foot [21].

#### 2.2.5. Autologous Leucocyte/Platelet/Fibrin Patch

It has been recognized that during the initial phases of wound healing, platelets and leucocytes are recruited to the wound site and release growth factors such as PDGF [1]. While treatment with individual growth factors has not been reported to be beneficial, better success has been found in developing products that contain living platelets and leucocytes as opposed to just individual growth factors. In these systems, platelets and leucocytes are harvested from patients and placed in a fibrin membrane for use on the patient’s wound. Encouragingly, products such as the 3C PATCH® (Reapplix) have shown good efficacy in diabetic foot ulcers [95,96,97], and this treatment has also been recommended by The International Working Group on the Diabetic Foot [21].

### 2.3. Pharmaceutical Drugs

#### 2.3.1. PDGF (Becapletmin)

Multiple growth factors have been identified as being critical in wound healing, including PDGF, EGF, FGF and TGF. However, only PDGF has been shown to augment wound healing in vivo [98]. PDGF is predominantly synthesized by platelets and is a dimer of A and/or B chains held together by disulfide bonds. There are three known isomers of PDGF that have been isolated from human platelets, AA, BB and AB, the most potent of which is BB. PDGF can bind to cells via two cell surface receptors: α-PDGF and β-PDGF. α-PDGF is a non-specific receptor while β-PDGF specifically binds PDGF-BB. β-PDGF is the most common receptor found in humans; as such, only PDGF-BB has been explored as a therapeutic [98].

Becaplermin is a homodimeric protein produced from DNA technology whereby the gene for the B chain PDGF is inserted into *Saccharomyces cerevisiae.* Becaplermin’s biological activity is like endogenous PDGF-BB specifically in its ability to promote chemotactic recruitment and proliferation of cells involved in wound repair. Becaplermin has been used extensively in the management of diabetic foot ulceration [99] and is currently licensed as Regranex^®^ (Smith and Nephew) [100]. Extensive animal and human studies have been carried out to demonstrate the efficacy of Becaplermin. Pierce et al. applied PDGF to incision wounds in rats and found that it both accelerated wound healing and improved the breaking strength of the wound [101]. The in vitro evidence of PDGF’s direct effect on keratinocytes is lacking however, it has been shown to increase the rate of epithelization in vivo. It is thus hypothesized that PDGF indirectly affects reepithelization through the recruitment of macrophages and fibroblasts [102]. Controversially, upon the original FDA approval of Regranex^®^ in 2008, it had a black box warning owing to an increased rate of mortality from secondary malignancy [103]. However, in 2018, the black box warning was removed after multiple studies including one by Ziyadeh et al., and showed that there was no increased incidence of cancer or cancer mortality associated with Regranex^®^ gel use [104].

#### 2.3.2. Phenytoin

Phenytoin (diphenylhydantoin) is a medication that was FDA approved in 1939 to treat convulsive disorders, such as epilepsy and seizures. However, according to the National Health Services (NHS), inflamed gums, specifically the development of fibrous overgrowth of gingiva and mild skin thickening, were common side effects of the use of phenytoin [105]. This stimulatory effect of phenytoin on connective tissues suggested its potential for use within wound care. Phenytoin has been shown to promote wound healing in dental extraction sockets [106] and corneal wounds [107]. Using a rat burn skin wound model, Sayar et al. showed administration of phenytoin increased healing through the development of vascularized, granulation tissue and increased collagen synthesis through re-epithelization [108]. Carneiro et al. conducted a clinical trial where acute burns were treated with topical phenytoin powder improved healing outcomes relative to Silverex, a cream containing SSD, commonly used for burn treatment [109]. Further, Inchingolo et al. investigated the use of topical phenytoin on bedsores by administering phenytoin-soaked patches at 12-h intervals. The patients treated with the phenytoin patches healed significantly quicker than those treated with water solution-soaked patches [110]. The exact mechanism of phenytoin in wound healing is unknown; however, studies suggest phenytoin promotes collagen deposition, decreases wound exudate and bacteria contamination [111,112] and may promote fibroblast proliferation [113].

#### 2.3.3. Vitamin A/Retinoids

Vitamin A is an essential fat-soluble dietary vitamin that is known to play a key role in epidermal maintenance by promoting desquamation and maturation through decreased production of keratin, keratohyalin granules and desmosomes. Within wound healing, vitamin A is known to contribute through the stimulation of angiogenesis, epithelization, and collagen synthesis. The pro-healing efficacy of vitamin A can rescue the antagonistic effect of steroids upon healing. However, their mechanism of healing is unknown. Vitamin A’s clinical use within wound care is hindered by its secondary effects. Systemic side effects include neurological and psychiatric effects and cutaneous effects include coarse hair, dry skin and widespread alopecia [114,115].

Owing to these side effects, retinoids were developed to combine the therapeutic effects of vitamin A with fewer adverse events. Retinoids are synthetic and natural derivatives of vitamin A. They bind to nuclear receptors on keratinocytes and regulate gene expression. First-generation retinoids, isotretinoin, all-trans-retinoic acid (tretinoin) and 9-cis-retinoic acid are nonaromatic compounds with modification on the polar end group and polyene side chain of vitamin A [116,117]. Originally an acne vulgaris therapeutic, retinoids have proven useful in preoperative facial rejuvenation and wound management [118,119,120]. Second-generation retinoids are monoaromatic formed by replacing the cyclic end of vitamin A with a modified ring. The most used is acitretin, which is used to treat psoriasis and other conditions which involve abnormal keratinization. Third-generation retinoids are polyaromatic compounds formed by cyclization of polyene side chains. Tazarotene is used for psoriasis and adapalene is used for acne vulgaris [118].

The evidence for the use of topical retinoids for use on wounds is conflicting. Tretinoin’s capacity to improve wound healing is thought to be due to its ability to reduce the production of procollagen in fibroblasts. This has been shown to be advantageous in hypertrophic scars and keloids. However, abnormal healing has also been reported through increased collagen degradation. Tom et al. showed a significant increase in diabetic ulcer healing in tretinoin-treated patients compared to placebo [121]. These findings were further supported by Paquette et al. in patients with chronic leg ulcers from venous disease or rheumatoid arthritis. With a short 10-minute application of 0.05% retinoic acid solution, within 7 days granulation tissue started to appear [122]. However, several studies have shown conflicting evidence against the utility of retinoids in wound healing. Watcher and Wheeland found that tretinoin resulted in significant retardation of reepithelization [123]. Overall, the evidence for good outcomes with retinoic acid in wounds with unfavorable baseline conditions is positive [114].

#### 2.3.4. Hypochlorous Acid

The immune system produces a range of ROS to protect from invading pathogens. During neutrophil activation, respiratory bursts generate H_2_O_2_ and activated granule enzyme myeloperoxidase converts H_2_O_2_ to hypochlorous acid (HOCl) in the presence of Cl^−^ and H^+^. HOCl causes cell death by oxidation of sulfhydryl enzymes and amino acids, ring chlorination of amino acids, loss of intracellular contents, decreased uptake of nutrients, inhibition of protein synthesis, decreased oxygen uptake, breaks DNA and depressed DNA synthesis [124,125]. As such, HOCl is a known antimicrobial capable of clearing bacterial, viral, and fungal contamination from the wound. Using an in vitro wound migration model, Sakarya et al. found that applying an HOCl solution, as an antimicrobial agent, in a dose-dependent manner increased keratinocyte and fibroblast migration [124]. Da Costa et al. showed that HOCl was effective in significantly increasing wound closure in a murine cutaneous wound model. They also showed that HOCl increased vascularization, increased neutrophil activity in the early phase of wounding and increased collagen [126]. Further work done by Dharap et al. showed success in the clinic where patients’ ulcers were dressed with Oxum, a super oxidized solution containing HOCl, reduced ulcer size and inflammation [127].

#### 2.3.5. Pentoxifylline

Pentoxifylline (PTX) is a dimethylxanthine derivative that increases cyclic adenosine monophosphate (cAMP) levels in the smooth muscle of blood vessels resulting in improved blood flow and oxygenation of ischemic tissues. It is also known to increase red and white cell filterability and platelet aggregation, fibrinogen levels and decrease whole blood viscosity [128]. This antithrombotic effect of PTX is linked to its induction of prostacyclin synthesis and inhibition of phosphodiesterase E enzyme. Prostacyclin is a potent vasodilator and platelet aggregation inhibitor. Further, PTX has been shown to inhibit the synthesis of inflammatory mediators, decrease cytokine release, suppress leukocyte function and reduce oxidative stress [129]. Velaei et al. used PTX as a treatment in a pressure wound-induced model on rats and reported accelerated wound healing through undefined mechanisms [130]. A study by Lim et al. tested PTX on a burn wound model, where relative to their small sample size, they did find a benefit compared to the placebo [131]. While the experimental data were mixed, Rawlins et al. showed in a clinical study that PTX was able to significantly improve perioral burns and improve scarring outcomes owing to PTX’s ability to inhibit fibroblast proliferation resulting in a decrease in type I and III collagens and glycosaminoglycans and increase collagenase activity [132]. Overall, further investigation is needed to define the success of PTX in wound care and the mechanisms by which it acts.

#### 2.3.6. Metformin

Metformin is an oral diabetic medication that helps lower blood sugar levels in type 2 diabetic patients. Interestingly, metformin treatment improved wound healing in aged rats, increasing both vascularization of the wound bed and proliferation of keratinocytes through activation of AMP-activated protein kinase (AMPK) [133]. Metformin has also been shown to boost M2 macrophage polarization through the induction of AMPK and mTOR and accelerate wound healing [134]. Further, Han et al. showed that metformin accelerated wound healing in the murine diabetic wound model [135]. While metformin is a promising therapeutic in diabetic patients due to its ability to treat the potential underlying pathology, further research is needed to assess its direct effects on wound healing.

## 3. Conclusions and Future Perspectives

The wound environment is a complex and changing environment, which creates unique challenges in the development of new and effective therapeutics. For example, treatments that promote an immune response may help prevent biofilm formation but could impede the transition from the inflammatory phase to the proliferative phase. Spatial differences also can complicate treatment approaches, particularly in keratinocytes, which need to migrate at the wound edges, but proliferate further away from the wound [136]. It is therefore essential to develop therapeutic strategies which allow for precise spatial and temporal drug release. An ideal delivery system would enable maximum therapeutic benefit by protecting the therapeutic payload from proteolysis, localizing bioavailability, and limiting systemic uptake and distribution to enable release maintenance at a physiologically relevant dose and duration. There is a range of biomaterials that can act as delivery vehicles including hydrogels, scaffolds and particles [137]. Hydrogels are the favored method of drug delivery in wound care owing to their multifaceted functionality. They provide a physical barrier between the wound and the external environment, preventing further pathogenic contamination, they are semi-permeable, allowing vapor transmission and oxygen and carbon dioxide exchange, and can be made of polymers that have intrinsic antimicrobial and pro-healing properties themselves such as chitosan. Importantly, the physical properties of hydrogels can be tuned to release the therapeutic at the optimal rate and concentration or in response to an environmental or physiological change, such as using H_2_O_2_ to trigger therapeutic release [138] or wound pH [139] to maximize efficacy [25].

Traditional therapeutics, such as aloe vera, have been used for centuries to treat wounds, and several natural products, including manuka honey and birch bark extract, are now sold commercially as a wound therapy. While progress has been made in the development of targeted therapeutics, the mechanisms of action of many of these treatments remain ill-defined. Contributing to the difficulty in developing new treatments is the fact that the underlying mechanisms regulating normal wound healing are still being elucidated (Table 1). This knowledge gap has made it difficult to pinpoint exactly what is dysfunctional in chronic wounds and how to correct these defects to normalize the wound environment. Compounding this problem, patients with chronic wounds often have underlying pathologies, like diabetes, which further impairs the healing process and creates an altered wound environment. Despite these challenges, recent technological advances in single-cell multi-omics and spatial profiling have vastly expanded our ability to interrogate the wound microenvironment and the dynamic interplay between cell populations during the wound healing process. These studies will undoubtedly lead to new, mechanism-driven treatments with the potential to attack root causes of dysfunctional wound closure and vastly improve patient outcomes. In summary, there are multiple therapeutic options available that stimulate wound healing to some extent, but there are substantial unmet needs highlighting the necessity for more effective treatments to improve the quality of life for individuals battling chronic wounds.

## Figures and Tables

**Table 1:** Overview of the different categories of pro-healing drugs covered in the review, the drug name, effect on healing and commercial name.

Category	Name	Effect on Wound Healing	Commercial Name
Natural Products	Antibiotics	- Antimicrobial	Varied
	Silver sulfadiazine	- Antimicrobial	N/A
	Medicinal Honey	- Upregulate proinflammatory cytokines - pH reduction - Antioxidative/ROS scavenging	MediHoney^TM^
	Curcumin	- Anti-inflammatory - Antioxidative/ROS scavenging - Increases collagen synthesis and production	N/A
	Aloe Vera	- Antioxidative/ROS scavenging - Stimulate fibroblast activity and proliferation - Anti-inflammatory - Upregulation of white blood cells	N/A
	Birch Bark	- Increases keratinocyte migration	Episalvan^®^
Human-derived Factors	MSCs	- Immunomodulation - Cell proliferation and differentiation - Promote production of growth factors data	N/A
	Collagenase	- Promotes keratinocyte proliferation - Promotes keratinocyte migration	Santyl^®^
Pharmaceutical Drugs	PDGF (Becapletmin)	- Promotes chemotactic recruitment - Promotes cellular proliferation - Increases macrophage and fibroblast recruitment	Regranex^®^
	Phenytoin	- Increases vascularization of granulation tissue - Increase in collagen synthesis	Dilatin
	Vitamin A/Retinoids	- Promotes angiogenesis - Promotes epithelization - Promotes collagen synthesis	Tretinoin
	Hypochlorous Acid	- Increases keratinocyte migration - Increases vascularization	N/A
	Pentoxifylline	- Improves blood flow and oxygenation - Increases platelet aggregation	Trental^®^
	Metformin	- Macrophage transition from M1 (pro-inflammatory) to M2 (anti-inflammatory)	Metformin

## Data Availability

No new data were created or analyzed in this study. Data sharing is not applicable to this article.
